# A Comparative Study of Serum and Follicular Fluid Leptin Concentrations among Explained Infertile, Unexplained Infertile and Fertile Women

**DOI:** 10.22074/ijfs.2015.4235

**Published:** 2015-07-27

**Authors:** Zahra Kamyabi, Tayebe Gholamalizade

**Affiliations:** 1 Department of Infertility, Afzalipour Hospital, Kerman University of Medical Sciences, Kerman, Iran; 2 Kerman University of Medical Sciences, Kerman, Iran

**Keywords:** Infertility, Leptin, Follicular Fluid, Serum

## Abstract

**Background:**

The relationship between metabolism and reproduction has been always considered as an important topic in female endocrinology. It seems that leptin is one of the involved factors in infertility. Leptin, in addition to regulating body weight plays an important role in regulation of endocrine, reproductive and immune systems. The aim of this stduy is to compare serum and follicular fluid leptin concentrations in order to find the role of leptin level in infertility.

**Materials and Methods:**

This case-control study was performed from September 2010 to March 2013. A total of 90 women referred to the Infertility Center of Afzalipour Hos- pital, Kerman, Iran, and divided into three equal groups (n=30/per group) of explained infertile (including 4 subgroups), unexplained infertile and normal fertile (control group). The three groups were matched in regard to demographic features [age: 20-40 years and body mass index (BMI): 20-25]. In order to determine leptin level, blood sample and fol- licular fluid were taken one hour prior and at the time of follicular puncture, respectively. Serum and follicular fluid leptin levels were measured using enzyme-linked immune sorbent assay (ELISA). Data were analyzed using descriptive-analytic tests, like Mann- Whitney and Kruskal Wallis tests, through Statistical Package for the Social Sciences (SPSS) version 16.

**Results:**

In explained infertile and fertile groups, as opposed to unexplained infertile group, mean leptin level was lower in follicular fluid than in serum. Mean follicular fluid leptin concentration in women with unexplained infertility was higher com- pared to the other two groups. Women with unexplained infertility had lower level of serum leptin in comparison to the other two groups. Follicular fluid leptin level in all subgroups of explained infertile group was lower as compared to unexplained and fertile women.

**Conclusion:**

The results suggested that high leptin level of follicular fluid is one of the main factors involved in infertility.

## Introduction

Infertility has been always addressed as one of the important, serious and costly health issues in different societies (1, 2). According to the previous studies performed in different countries, about 10-15% of couples suffering from infertility regard this disability as the worst experience in their life (3-5). This problem might cause marital conflicts, social injuries, divorce and other psycho-social problems (2, 6). At present, in Iran, about 3 million couples suffer from infertility. In 7% of infertile couple population, the cause of infertility is unknown.

The relationship between metabolism and reproduction has been always considered as controversial issues in the field of female endocrinology. Insulin, amino acids and low molecular weight IGF-binding protein-1 (IGFBP-1) have been introduced as effective signals in alterations of body fat and body mass index (BMI), but these alterations have been recently attributed to leptin level (7). Leptin, in addition to regulating body weight, plays an important role in regulating the functions of endocrine, reproductive and immune systems through suppressing food intake and increasing energy consumption. Deficiency of leptin or its receptors, in addition to causing obesity, leads to disturbing reproductive cycle, hormonal imbalance, as well as disorders of immune system, hematopoietic system and bone metabolism (8). These observations have indicated the important role of leptin in several physiologic processes and the relationship between abnormal leptin levels and many disorders (9).

Leptin affects menstrual cycles, directly and indirectly. It have been reported that leptin directly affects ovaries and hypothalamic-pituitary axis. Furthermore, its effect on follicle stimulating hormone (FSH)-dependent estradiol production in animals and its role in preventing starvation-induced delay in ovulation in mice show indirect effect of leptin on the luteinizing hormone (LH) concentration (10).

Leptin is an adipocyte hormone acting as a link between adipose tissue and reproductive system. It is also considered as a type 1 cytokine, due to its role in cell growth and maturation (11). Recent studies have reported that leptin is produced by both granulosa and cumulus cells of ovarian follicles (10, 11).

Leptin is used in the treatment of hypoleptinemia due to energy deprivation state, leptin deficiency and obesity-related hyperleptinemia. Due to leptin resistance in some obese individuals, leptin treatmentis used in patients with complete or relative leptin deficiency including patients with hypothalamic amenorrhea and lipoatrophy, but coadministration of this hormone with leptin sensitizers has been resulted in better outcomes in the treatment of obesity (8).

Since it has been proved that there is a definite relationship between infertility and menstrual irregularities in women with abnormal obese (OB) gene expression and peritoneal fluid is also known as an active biologic environment that is essential for regulation of ovarian function, ovulation, zygote implantation, and follicle collection (9, 11), any change in the concentration of substances in this environment is likely to affect ovarian function. Furthermore it can be postulated that the origin of some substances like leptin might be from follicular fluid.

The aim of this study was to compare serum and follicular fluid leptin concentrations among explained infertile women, unexplained infertile women and fertile women in order to find the role of leptin level in infertility. In the present study, without applying any invasive method more than the treatment process, three groups were compared in regard to serum and follicular fluid leptin concentration using blood sample and follicular fluid of assisted reproductive technology (ART) candidates.

## Materials and Methods

In this case-control study, 90 women using convenient sampling method with regard to the inclusion criteria referred to the Infertility Center of Afzalipour Hospital, Kerman, Iran. After ensuring, all personal information remained confidential and there was no intervention with their treatment process. This study approved by Ethical Committee of Kerman University of Medical Sciences and done from September 2010 to March 2013 and all participants provided an informed consent.

Inclusion criteria were as follow (the presence of all 7 criteria was necessary): i. Age between 20 to 40 years, ii. BMI between 20 to 25 kg/m_2_, iii. Normal levels of FSH, LH (on days 2 and 3 of menstrual cycle), prolactin, testosterone and progesterone (between days of 19 and 21of menstrual cycle), iv. Normal semen fluid analysis and pelvic ultrasonography, v. Absence of any underlying complex disorders like diabetes, obesity, cardiovascular disease, any type of metabolic diseases and malignancies, vi. Negative result of autoantibody test and vii. No use of anti-inflammatory medicines. It should be mentioned that only infertile participants underwent laparoscopy and hysterosalpingography (HSG). First the information regarding demographic and clinical characteristics were collected and infertility investigations, including ovulation state, cervical and uterine factors, and patency of fallopian tubes, were then performed in couples referred for *in vitro* fertilization (IVF). All partners had normal sperm analysis. If infertility was due to male factor, the case was excluded from our study because they had no history of pregnancy and were not considered as unexplained infertility.

Subjects were divided into three groups of explained infertile group, unexplained infertile group and fertile group. The explained infertile group (n=30) contained women with one or more infertility factors. This group was divided into the four subgroups including cervical, endometrial, tubal and peritoneal factors based on medical history and findings of physical exam, pelvic ultrasonography, HSG or laparoscopy. The unexplained infertile group (n=30) contained women with unknown causes of infertility. The normal fertile group (n=30), as control group, contained normal fertile women referred for oocyte donation who had regular menstrual cycles with normal fertility factor.

In all groups, the long agonist protocol for controlled ovarian hyperstimulation (COH) was used. Briefly, COH was performed by administration of human menopausal gonadotrophin (HMG, Ferring, Germany) after pituitary suppression with buserelin (superfact, Aboureyhan, Iran), starting in the midluteal phase of the preceding cycle. The dosages of gonadotropins were individualized according to serum estradiol (E_2_) levels and transvaginally ultrasonic measurements of the follicles. When at least three follicles reached to diameter of 16-18 mm, ovulation was induced by the administration of 10,000 IU human chorionic gonadotropin (hCG) 36 hours before puncture. Average number of follicles of each case was 12 (9-16).

In each patient before oocyte aspiration, a peripheral blood sample was taken from antecubital vein one hour prior to puncture. Blood samples were transferred into sterile tubes. Then under general anesthesia (same protocol), a surgeon performed the ultrasound-guided transvaginal oocyte aspiration using a 16-17 gouge long needle. The follicular fluid samples were carefully collected from the first aspirated follicle of each ovary, and the follicular fluid samples without visible blood contamination were used in this study. The oocyte retrieval was continued for all IVF candidates. Blood and follicular fluid samples were immediately centrifuged at 3000 rpm for 10 minutes and the supernatants were stored at -70˚C for further analysis.

To measure the serum and follicular fluid leptin concentrations, enzyme-linked immunosorbent assay (ELISA) kit (Labor Diagnostica Nord GmbH, Germany) was used. All measurements were carried out in duplicate. The intra- and inter- assay coefficients of variation were less than 3.7 and 6.8%, respectively, with the standard range of 0.5-100 ng/ml.

### Statistical analysis

To present descriptive statistics, mean ±standard deviation (SD) was used. In order to compare follicular fluid and serum leptin concentrations among three groups, Kruskal Wallis test was used. To compare follicular fluid and serum leptin concentrations within groups, Mann Whitney test was used. All statistical analyses were performed throughthe Statistical Package for the Social Sciences (SPSS, SPSS Inc., Chicago, IL, USA) version 16 and P value less than 0.05 was considered as statistically significant.

## Results

In the present study, 90 women including 30 explained infertile women (group 1), 30 unexplained infertile women (group 2) and 30 fertile women (group 3) were studied. Demographic and clinical data regarding subsequent IVF of these groups are shown in table 1. The age and BMI displayed no significant differences among three groups. Progesterone, testosterone, FSH and LH levels were not significantly different (P>0.05 for all). The number of transplanted embryos demonstrated no significant difference. The percentage of fertility rate and good quality embryos in unexplained infertile group were lower in comparison to explained and control groups, but differences were not significant (P>0.05).

In explained infertile group, mean follicular fluid leptin level (19.92 ± 17.87) was lower than mean serum leptin level (33.13 ± 17.31), but the difference was not significant (P=0.11). In unexplained infertile group, mean follicular fluid leptin level (48.9 ± 20.20) was significantly (P<0.001) higher than mean serum leptin level (27.83 ± 25.29).

**Fig.1:**
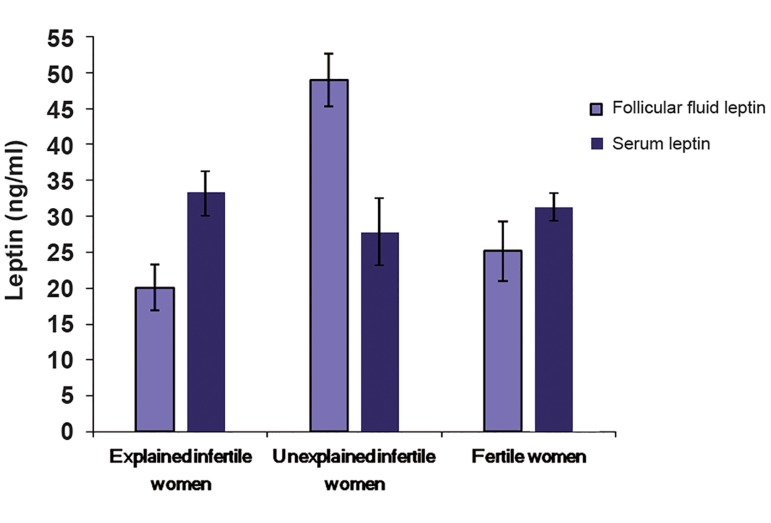
Comparison of leptin concentration in follicular fluid and serum of three studied groups (n=30/per group). Data are presented as mean ± SD.

In fertile group, mean follicular fluid leptin level (25.07 ± 22.36) was lower than mean serum leptin level (31.27 ± 11.02), but the difference was not significant (P=0.19,Table 2). Mean follicular fluid leptin levels showed a significant difference within the groups (P<0.001). Among the groups, mean follicular fluid leptin level was higher in unexplained infertile women (group 2), fertile women (group 3) and explained infertile (group 1), respectively. In regard to mean serum leptin, explained infertile women had higher mean serum leptin level (33.13 ± 17.31) compared to unexplained infertile women (27.83 ± 25.29) and fertile women (31.27 ± 11.02), but the difference was not significant (P=0.070, Fig.1).

Follicular fluid and serum leptin levels in the each subgroup of explained infertile women were determined and the results were compared with unexplained infertile and fertile woman. Follicular leptin level in all explained infertility subgroups was significantly lower in comparison to unexplained and fertile groups (P<0.001), but serum leptin level in the explained infertile subgroups showed no significant difference as compared with unexplained infertile and fertile groups.

**Table 1:** Demographic data and clinical characteristics


	Median (range)			
	Unexplained infertilewomen (n=30)	Explained infertilewomen (n=30)	Fertilewomen (n=30)	Significance

**Age (Y)**	30 (20-38)	31(25-40)	29 (20-38)	>0.05
**BMI (kg/m^2^)**	24.84 (20.08-24.34)	23.38 (21.01-24.58)	22.26 (20.17-24.71)	>0.05
**No. of follicles >14 mm (after COH)**	12 (7-14)	13(9-15)	12 (7-15)	>0.05
**No. of oocytes**	8 (7-10)	10 (6-13)	9 (7-14)	>0.05
**Fertility rate %**	76.90	78.80	79.75	>0.05
**Good quality embryo (%)**	68.20	73.92	75.60	>0.05


No; Number, BMI; Body mass index and COH; Controlled ovarian hyperstimulation.

**Table 2:** Leptin concentration in serum and follicular fluid of three studied groups


	Leptin level		
Group	Follicular fluid	Leptin levelSerum	P value*

Explained infertile	19.92± 17.87^&^	33.13± 17.31^¥^	0.110
Unexplained infertile	48.90± 20.20^#^	27.83± 25.29	<0.001
Fertile	25.07± 22.36	31.27± 11.02	0.19
P value**	<0.001	0.07	


*; Mann-Whitney test, **; Kruskal-Wallis test, ^&^; There is a significant difference compared to unexplained infertile group, ^¥^; Data are shown as mean ± standard deviation and ^#^; The unit of measurement is ng/ml. P<0.05 is considered statistically significant.

## Discussion

In the current investigation, follicular fluid leptin level in unexplained infertility group was more than that in explained infertility and normal fertile groups.

Infertility has been always addressed as one of the important, serious and costly health issues in different societies (1, 2). For this, various studies have already been performed to discover the causes of infertility, among them high leptin level has been considered as one of the important and effective factors in several studies (12).

Leptin has a wide range of functions from acting as an anti-obesity factor to an effective factor in reproduction, hematopoiesis, angiogenesis and T lymphocytes system (17).

Some investigators have suggested that leptin might exert a double role in regulation of reproduction. They showed that when leptin level is lower than normal, it can exert a negative effect on endocrine system, regulating reproduction, while when leptin level is higher than normal, it negatively affects normal function of ovary and fetus development (18, 19).

The present study suggested that high level of follicular fluid leptin has a negative effect on reproduction in unexplained infertility. Enhanced leptin level may inhibit aromatase activities and prevent the transformation of androgen to estrogen, leading to the elevation of serum androgen and interferers with ovarian follicle growth and ovulation by suppressing estrogen production (20).

A recent study revealed that serum leptin level is significantly higher in unexplained infertile women compared to the fertile group and suggested that leptin as cytokine-like or hormone affects pathophysiology of infertility, but due to study limitation, leptin levels in serum and peritoneal fluid were not compared (19).

In a study with Takeuchi and Tsutsumi (21), serum leptin level in unexplained infertility group was higher compared to polycystic ovary syndrome (PCOS) group, but difference was not significant, whereas in a study with Demir et al. (22), comparison of serum leptin levels in unexplained infertile woman and fertile woman demonstrated significant higher serum leptin levels in unexplained infertile woman. However, in the present study, unexplained infertile group had lower serum leptin level in comparison to the explained infertile and fertile groups, but the difference was not significant. It seems that further studies are required to clarify this point.

Gogacz et al. (10) have reported a significant increase in peritoneal fluid leptin level in endometriosis and unexplained infertility. They had no control group for comparison and suggested similar studies containing a control group. They also suggested peritoneal fluid leptin might be originated from follicular fluid. To consider the mentioned study and association between the increased level of peritoneal leptin with endometriosis and unexplained infertility, it can be proposed that leptin stimulates toxic factors in peritoneal fluid and also decreases the quality of oocyte in endometriosis. It should be noted that the cause of unexplained infertility needs to be identified with new advances in infertility treatment and leptin is likely to be considered as one of the involved factors.

Another study represented that peritoneal fluid leptin level was significantly higher in unexplained infertile group compared to patients with PCOS. In the mentioned study, serum leptin level was also higher in unexplained infertile group but not significantly (22).

The results of our study showed significant higher follicular fluid leptin level in unexplained infertile women compared to the explained infertile and fertile groups, but serum leptin level in this group was insignificantly lower than the explained infertile and fertile groups. Fertility rate and number of good quality embryos in unexplained infertile group were lower in comparison to explained and control group, but there were no significant differences. It seems that further studies are required to establish effect of high follicular leptin level on infertility. It seems that systemic effects of leptin in blood flow differ from its local effects in follicular fluid as it was observed in the present study that unexplained infertile group had higher follicular fluid leptin level and lower serum leptin level in comparison to the other two groups. Whether these effects act independently or not is a point deserving attention.

This relationship presents differently in persons with different BMI; for example, in patients with anorexia and low BMI and in obese individuals with high BMI, leptin shows contradictory effects. It can be hypothesized that a specified concentration of leptin is required for female productivity and both high and low levels of leptin can affect fertility. This effect is seen for both systemic leptin and follicular and peritoneal leptin (18).

In another study, systemic and central effects of leptin on gonadotropins have been less accepted and leptin effect on ovary has been considered more. There are more leptin receptors on ovary than in central nervous system (CNS) (19).

## Conclusion

The obtained results showed that high leptin level in follicular fluid affect unexplained infertility. Therefore, our finding suggested that high leptin level of follicular fluid is one of the main factors involved in infertility. The fact that whether leptin acts independently or as an associated factor requires more studies.
